# Patterns and predictors of help-seeking intentions for suicidal ideation compared to other health conditions among rural Chinese adults

**DOI:** 10.1186/s12888-024-06186-0

**Published:** 2024-10-24

**Authors:** Yang Wu, Zhenzhen Chen, Yaoguang Guo, Jin Han

**Affiliations:** 1 Huazhong University of Science and Technology, Wuhan, Hubei Province People’s Republic of China; 2 Central China Normal University, Wuhan, Hubei Province People’s Republic of China; 3 Guangzhou Medical University, Guangzhou, Guangdong Province People’s Republic of China; 4 New York University Shanghai, Shanghai, People’s Republic of China

**Keywords:** Psychological help-seeking, Suicidal ideation, Rural residents

## Abstract

Psychological help-seeking for suicidal ideation is a critical aspect of effective suicide prevention. Past research has documented low help-seeking amongst rural residents in China. This study investigates the patterns and predictors of help-seeking intentions for suicidal ideation versus mental (depression) and physical (heart disease) health conditions among Chinese rural residents using General Help-Seeking Questionnaire Vignette version. A total of 143 rural participants from sixteen villages in Hubei province of China completed the survey via home-visiting interviews. Results revealed a general trend that the help-seeking intentions decreased as the helping sources shifted from close others (spouses, family, etc.) to professional helpers and online sources. Additionally, rural residents with higher educational levels, high self-efficacy, and more severe suicide ideations were more willing to seek help; and suicide literacy was found to be negatively associated with help-seeking intentions. The implications of the research findings are discussed.

**Supplementary Information:**

The online version contains supplementary material available at 10.1186/s12888-024-06186-0.

## Introduction

Suicide is a leading cause of unnatural death among the adult population globally [10]. According to WHO statistics [54, 55], each year more than 700,000 cases of suicide are reported across the world. Although China had once ranked as the country with the highest rates of suicide in the world, suicide rates in China exhibit an overall downward trend in the past two decades [27, 35, 62] potentially due to the conjoint influence of multiple factors, including economic growth, improvement in healthcare and mental health awareness.

However, against the backdrop of the overall declining trend of suicide rates, rural areas in China still reported relatively high suicide rates. Some reports show that suicide rates in rural areas could be two or three times higher than in urban areas [35, 48]. Compared with urban areas, rural areas in China have relatively poorer economic conditions and limited coverage of social welfare services, and rural residents generally received less education than their urban counterparts and were more influenced by traditional cultural norms; relatedly, rural healthcare condition is much more limited, with indices such as accessibility of mental health services and mental health literacy all lagging far behind domestically [36, 37]. This broad range of economic, ecological, systemic as well as cultural factors may all have contributed to the elevated suicide rates in rural areas [15, 17, 19, 25]. The overall poor mental health awareness (and the various mental health stigmas, and suicide stigmas that follow) and the relatively low psychological help-seeking intention could be crucial proximal contributors to high suicide rates among them [60].

Psychological help-seeking for suicidal ideation is a vital part of suicide prevention. The general view is that suicide is a result of the myriad environmental and individual factors [29, 33, 45, 57]. A complex constellation of intrapsychic processes, especially the sense of hopelessness, psychological pain, and the associated cognitive processes, are often the direct precursors to suicide attempts [9, 32, 53]. Given this, timely help-seeking immediately before or after the emergence of suicide ideation could help mitigate or ameliorate these cognitive and emotional processes that may lead to active suicidal attempts [20, 21, 23, 30], which may ultimately reduce overall suicidal behaviors. However, data from diverse sources showed that people with suicide ideation or a history of suicide attempts are reluctant to seek help [43, 52], and rural residents often reported even lower help-seeking intentions and behaviors than urban residents [48]. Moreover, studies found that seeking help from professional channels is more efficacious than from non-professional ones (e.g., family, friends, netizens) [42]. Yet currently study is limited on whether rural residents could differentiate on the varying helping sources and whether this may have contributed to low help-seeing rates in rural areas.

Recently, scholars have proposed several theories to elucidate the psychological processes underlying psychological help-seeking for suicidal ideation, such as the Theory of Planned Behavior [1, 3], three-stage model for mental health help-seeking [14], Andersen model of health care utilization [4, 5], and various integrated models [34, 40]. Combining these different theoretical frameworks, factors that may affect suicide help-seeking could be categorized into three types: (1) Predisposing characteristics (e.g., demographics), (2) Need factors (i.e., to impel one to realize that there is a problem and feel the need to seek help), and (3) Enabling resources (i.e., the available resources that could “enables” the help-seeking).

First, among the predisposing characteristics, demographic variables such as gender, age, and educational levels have received considerable research attention, yet related research often yielded mixed results. For example, some studies found that females are more likely to seek help than males, partly because of the masculinity ideal of men, such as self-reliance and toughness, under the influence of which men tended to perceive psychological help-seeking as showing weaknesses [12, 20, 21, 23]. However, another line of research noticed that among medically serious suicide attempters in rural China, the likelihood of help-seeking is actually higher in men than in women, and this could stem from the weaker social network of women in rural environments than men, as well as the remnants of patriarchal culture in rural areas that restricted female agency [48]. Similarly, while some studies found that older people have a stronger intention to seek help [12], other studies showed otherwise: it is the younger generation that is more willing to seek help [20, 21, 23]. In most studies, educational levels could positively predict help-seeking [20, 21, 23, 60, 63], probably because educational experiences may increase mental health literacy and reduce stigma. Yet in a study that investigated psychological help-seeking among adults in rural China, educational levels were found negatively related to help-seeking [61]. A recent study conducted in Japan showed similar results [41].

Second, among the need factors, individuals’ mental health conditions, suicidal ideation and/or behavior, exposure to suicide, as well as knowledge of suicide prevention known as suicide literacy are the most studied in the literature. It is believed that the need for help-seeking would arise as individuals become aware of their discomforts when their mental health condition worsens and suicidal ideation increases [2, 20, 21, 23]. In addition, only when people have an accurate self-appraisal of suicidal ideation can they perceive the need for help-seeking. During this process, prior exposure to suicide may help raise awareness of the suicide issue, enhance the ability to detect early symptoms of suicide ideation, and thus potentially increase help-seeking behaviors [12, 31]. A similar effect could be found in suicide literacy. Suicide literacy typically comprises factual knowledge about suicide and effective coping strategies. Individuals with higher suicide literacy could better identify their need for help and could recognize the value of psychological help-seeking, which may translate into greater levels of help-seeking intention and behaviors [20, 21, 23]. For example, Calear et al. [12] showed that suicide literacy could positively predict favorable attitudes toward help-seeking for suicidal ideation, and the finding was replicated in many countries [61, 63]. However, in a study of university students from China and Australia, the relationship between suicide literacy and help-seeking is not significant [20, 21, 23].

Third, enabling resources could be understood as a miscellaneous set of factors that may affect people’s willingness to seek help and their actual help-seeking behaviors, when they have and are already aware of the need for help-seeking. Previous research has generally explored the effects of external resources or objective conditions of the environment, such as economic conditions, medical insurance status, and availability of mental health services across the areas [4, 34]. In this regard, the ratio of psychiatrists to the population is low in China, the availability and accessibility of mental health services is limited, and these are especially pronounced in rural areas [46, 48, 61], which could all negatively affect psychological help-seeking for suicidal ideation. However, in a similar or the same area (i.e., with same or similar external conditions), the individual differences among help-seeking behaviors would then have to be sought in those enabling resources that are internal to an individual. One such internal factor that warrants investigation is one’s self-efficacy.

Self-efficacy refers to a set of cohering beliefs about “one’s capabilities to achieve specific goals or performance” [6, 51] in different types of activities. Previous research has shown that self-efficacy is closely associated with a variety of healthy behaviors and self-regulating behaviors [38]. Aside from acknowledging the need to seek help, an effective psychological help-seeking further requires an individual to search, screen, and select the helping sources according to his or her conditions, and after that, to take the time and effort to reach out to the chosen helping source. Compared with people with high self-efficacy, those with low self-efficacy would find these tasks more formidable and thus exhibit lower levels of both willingness to seek help and help-seeking behaviors [11]. Although recent studies have investigated the relationship between self-efficacy and general psychological help-seeking and self-help [38, 49], empirical data on the link between self-efficacy and help-seeking for suicidal ideation in rural residents remains limited.

To date, there is a dearth of studies on psychological help-seeking for suicidal ideation in rural China. The purpose of the present study is to fill this lacuna by examining the patterns of help-seeking intentions in rural communities and exploring its potential predictors. Specifically, given the higher rate of suicide in rural communities, this study aims to investigate the psychological help-seeking intentions among rural residents in China, with a special focus on whether the help-seeking intentions may differ between types of health conditions (suicide vs. mental disorder vs. physical disease) and among types of helping sources. In addition, the study also aims to explore the various influencing factors to help-seeking in rural areas from the perspective of tripartite classification of factors, i.e., the predisposing, need, and enabling factors. Specifically, we explored several demographic factors (age, educational levels, and gender) as “predisposing” factors; several mental health indices (psychological distress, depression, anxiety, suicide ideation), suicide exposure, and suicide literacy were explored as potential “need” factors; and last we chose self-efficacy as a proxy for enabling resources in the present study. As this study is largely exploratory, we only hypothesized that rural residents in China may differ in their help-seeking intentions across helping sources and across health conditions.

## Method

### Measurement

#### General help-seeking intentions

Help-seeking intentions for different types of problems were measured by an adapted form of the General Help-Seeking Questionnaire Vignette version (GHSQ-V; [56]). In its original form, participants were presented with seven vignettes containing validated symptomatic descriptions of 7 different problem types (stress, anxiety, depression, suicidal ideation, substance misuse, psychosis, and heart disease) and were asked to rate their help-seeking intentions towards a list of helping sources. In the present study, we chose three problem types, Depression, Suicidal Ideation, and Heart Disease. Heart disease is the only physical condition among the original seven vignettes in GHSQ-V and was chosen as a benchmark for comparison; depression is a common mental health disorder that has been found to be closely associated with risks of suicide. For each problem types the participants will read a vignette depicting a randomly named person suffering from its typical symptoms. The content of the vignette was drawn partly from Wilson et al. [56] and was translated into Chinese under the permission of and consultation with its original authors. A bilingual professional psychiatrist checked the vignettes for their accuracy both in translation and depiction. Participants were asked to read each of the vignettes before rating the likelihood that they would seek help from 10 help sources if they were the person depicted, namely, (1) their intimate partner (spouses), (2) friends, (3) parents, (4) other relatives, (5) psychological counselors, (6) doctors (in the hospital), (7) helplines, (8) websites, (9) online help-groups, and (10) social media. For each vignette, participants were also asked about the likelihood that they “would not seek help from anyone”. All ratings were made on a 4-point Likert scale (1 = “*Extremely unlikely*” to 4 = “*Extremely likely*”) rather than originally 7-point scale to ease the burden of responding for the rural participants. In addition, an open-ended question was added at the end of each vignette rating that allows participants to express what they think is the problem. The analysis of the open-ended question will be reported in a separate article and thus was omitted in the present study.

#### Depression

We adopted the Chinese version of the 9-item Patient Health Questionnaire-9 (PHQ-9) to measure the depression levels of participants [59]. Each of the PHQ-9 items describes a symptom that corresponded to one of the criteria for diagnosing depression in DSM-IV, such as “little interest or pleasure in doing things”. Participants were asked to rate the frequency with which they experienced the symptoms in the past two weeks using a 4-point Likert scale (0 = *Not at all*, 1 = *several days*, 2 = *more than half the days*, to 4 = *nearly every day*). The sum of the scale items constitutes the final depression score. In the present study, the reliability of PHQ-9 was satisfactory, with Cronbach’s α = 0.824.

#### Suicide ideation

A single item chosen from the PHQ-9 was used as an indicator of participants’ recent suicide ideation, “thinking that you would be better off dead or that you want to hurt yourself in some way”. We recorded their responses on the 4-point Likert scale (0 = *Not at all*, 1 = *several days*, 2 = *more than half the days*, to 4 = *nearly every day*) into a binary variable, such that people who have scored nonzero on the item were coded as 1 (“*recently having suicide ideation*”), and those who score zero remained 0 (“*no suicide ideation*”).

#### Anxiety

The Chinese version of 7-item General Anxiety Disorder-7 (GAD-7; [24, 47, 50]) was employed to measure participants’ anxiety. Past research has established that GAD-7 is an efficient and effective tool for screening anxiety disorder. The seven items of the scale were each describing a typical symptom of general anxiety disorder, such as “feeling nervous, anxious, or on edge”. Participants rated the frequency with which they experienced the symptoms in the past two weeks using a 4-point Likert scale (0 = *Not at all*, 1 = *several days*, 2 = *more than half the days*, to 4 = *nearly every day*). The sum of the scale items constitutes the final depression score. In the present study, the reliability of GAD-7 was satisfactory, with Cronbach’s α = 0.881.

#### Suicide literacy

The 11-item short form of the Literacy for Suicide Scale (LOSS-Short) was adopted to assess participants’ suicide literacy [7, 13]. The Chinese version was translated and validated by Han et al. [22]. The LOSS-Short Chinese version contains 11 statements regarding suicide, and participants responded on a 3-point scale (“true”, “false”, and “I don’t know”). An item example is “A suicidal person will always be suicidal and entertain thoughts of suicide” (False). Participants’ total number of correct answers served as the final literacy of suicide score.

#### Suicide exposure

Participants’ exposure to suicide was measured by a 10-item multiple choice scale [22, 26, 58]. The ten items of scale were each depicting a scenario of growing levels of suicide exposure, ranging from “no exposure” (0) to “respondent attempted suicide” (9). Participants were asked to check all items which they have experienced. The maximum level of exposure was the final score of the scale, ranging from 0 to 9.

#### Self-efficacy

Participants’ self-efficacy beliefs were assessed by a 10-item Generalized Self-Efficacy Scale [44]. The scale was originally developed in German as a 20-item version and was later shortened to 10 items. The Chinese version was translated and validated by Schwarzer et al. [44]. The scale contains 10 statements, such as “I can always manage to solve difficult problems if I try hard enough”. Participants rated their agreement with the items on a 4-point Likert scale, ranging from 1 (“*Not at all true*”) to 4 (“*Exactly true*”). The average score was the final score for the scale. In the present study, the reliability was satisfactory, with Cronbach’s α = 0.888.

#### Psychological distress

Distress Questionnaire-5 (DQ-5) was adopted to measure participants’ psychological distress [8]. Because DQ-5 has not been translated into Chinese, we first translate and back-translate the scale into Chinese under the permission of and consultation with its original authors. The Chinese version was validated in an additional college student sample (*N* = 499) (Han, Wu, Batterham, Shou, & Li; manuscript in preparation) and the validity and reliability were high, with Cronbach’s α = 0.823 and the confirmatory factor analysis showing a good fit (χ^2^ = 4.245, *df* = 5, *p* = 0.515, GFI = 0.997, RMSEA < 0.001). The average score was the final score for the scale. In the present rural sample, the reliability of DQ-5 is also satisfactory (Cronbach’s α = 0.809).

#### Demographics

Age was recorded as participants’ year of birth and was later recoded into age numbers. Gender was measured as male or female. Educational levels were measured by a complete eleven levels of different educational status to cover all possibilities, ranging from illiterate, literate but unschooled, to graduate school and beyond. Given that we found that half of the levels were sparse in the present sample, we reclassified the responses into four ordinal categories, (1) less than junior high school, (2) junior high school, (3) high school or equivalent, and (4) college or higher.

### Participants and procedure

Participants were randomly chosen from 16 villages or rural communities from four cities in Hubei province. The sampling was performed with the aid of geographical information systems (GIS) and global positioning system (GPS), to achieve a balanced design of sampling sites in terms of representativeness and population density. All the interviewers were graduate students from Huazhong University of Science and Technology, who had received professional training on face-to-face interviews and surveying techniques. We also set up a two-day warmup session before the interviewing began, to familiarize the interviewers with the aim, the structure, and the contents of the questionnaire. In most cases, each participant was interviewed individually by a trained interviewer in a private condition at their home. For participants who were aged, with heavy dialect, or felt uncomfortable being alone with a single interviewer (or vice versa), the field supervisor would send another interviewer to assist with the interviewing. The interviewers read the scale items to the participants, made sure they correctly understood the contents, and then recorded their oral answers. Upon completion, participants will receive a small gift (grocery valued at 30–40 yuan/person) and be thanked. The interviewing of participants took place in July 2019. All interviews were completed in approximately 30 to 40 minutes.

### Statistical analysis

To examine the help-seeking intention for different problem types and from different sources of helper, we first conducted a 3 (problem type) × 10 (helper) two-way within-subject design analysis of variance (ANOVA) and a one-way within-subject ANOVA on the effect of problem types on the participants’ tendency of not seeking any help.

To further examine whether factors associated with participants could affect their willingness to seek help and not to seek help, we tested two separate mixed models with random intercepts to account for the within-subject and between-subject variables. The Level 2 predictors of the two models were identical, which include participants’ demographics (e.g., gender, age, educational levels), mental health indicators (depression, anxiety, and psychological distress), self-efficacy, levels of suicide exposure, suicidal ideation, and suicide literacy. The Level 1 predictors for Model 1 (help-seeking intentions across helpers and problem types) include helpers and problem types, both within the subject, while predictors for Model 2 (the intention of not seeking help) only include problem types.

ANOVAs were performed on SPSS 22.0, and the other analyses were performed using R (version 4.4.1).

## Results

### Demographics and descriptive statistics

After deleting 21 incomplete cases, the total valid sample consisted of 143 participants, with 65 males, 75 females, and 3 missing (the gender of three participants was lost due to the investigator’s recording omission). It should be noted that considering the sensitive nature of suicide exposure, when respondents appear unwilling to answer questions related to suicide exposure, the interviewers will not ask follow-up questions. Thus, only 112 participants completed the suicide exposure scale, making a small fraction of analyses that involved suicide exposure restricted to this smaller sample. The other analyses were conducted on the full sample of 143 participants. The average age of the participants was 43.252 (*SD* = 15.478). A series of t-tests on all major variables showed that no significant difference existed between people who have and have not finished the suicide exposure scale.

The demographic characteristics of participants are described in Table 1. The descriptive statistics of the major variables are summarized in Table 2.

**Table 1:** Demographic characteristics of the rural sample

	*N*	Percentage
Complete sample	143	100%
Gender		
Male	65	45.5%
Female	75	52.4%
Missing	3	2.1%
Marital status		
Unmarried	26	18.2%
Married	111	77.6%
Widowed	2	1.4%
Divorced	1	0.7%
Refused to answer	2	1.4%
Missing	1	0.7%
Educational levels		
Less than junior high school	39	27.3%
Junior high school	34	23.8%
High school or equivalent	36	25.2%
Vocational College or above	33	23.1%
Missing	1	0.7%
Suicide Ideation		
Yes	10	7.0%
No	133	93.0%

**Table 2:** Descriptive statistics for variables of interest

	Mean	SD
DQ-5 (Distress)	1.726	0.682
PHQ-9 (Depression)	4.573	4.180
GAD-7 (Anxiety)	2.524	3.331
Suicide exposure	1.929	2.958
Suicide literacy	4.888	1.873
Self-efficacy	2.661	0.666
GHSQ-V		
**Depression**		
Spouse	3.182	0.932
Friends	2.706	0.948
Parents	2.615	1.100
Relatives	2.287	0.976
Counsellors	2.378	1.020
Doctors	1.965	0.930
Helplines	1.699	0.864
Websites	1.874	0.941
Help-groups	1.825	0.952
Social media	1.734	0.880
“Won’t seek help”	1.818	0.969
**Suicide Ideation**		
Spouse	2.993	1.058
Friends	2.643	1.010
Parents	2.490	1.156
Relatives	2.308	1.002
Counsellors	2.427	1.024
Doctors	2.196	0.981
Helplines	1.790	0.838
Websites	1.937	0.944
Help-groups	1.818	0.909
Social media	1.727	0.857
“Won’t seek help”	1.937	1.050
**Heart Disease**		
Spouse	3.203	0.939
Friends	2.622	0.977
Parents	2.699	1.014
Relatives	2.280	0.952
Counsellors	2.315	0.996
Doctors	3.084	0.968
Helplines	1.790	0.879
Websites	1.979	1.003
Help-groups	1.825	0.959
Social media	1.762	0.903
“Won’t seek help”	1.629	0.861

*DQ-5* Distress Questionnaire 5, *GAD-7* General Anxiety Disorder-7, *PHQ-9* Patient Health Questionnaire-9. PHQ-9, GAD-7 and suicide literacy were calculated as sum score, while DQ-5 and self-efficacy was mean score. Suicide exposure was calculated as a person’s highest level of exposure

### Help seeking intention

#### General pattern of help-seeking intentions

**Fig. 1:**
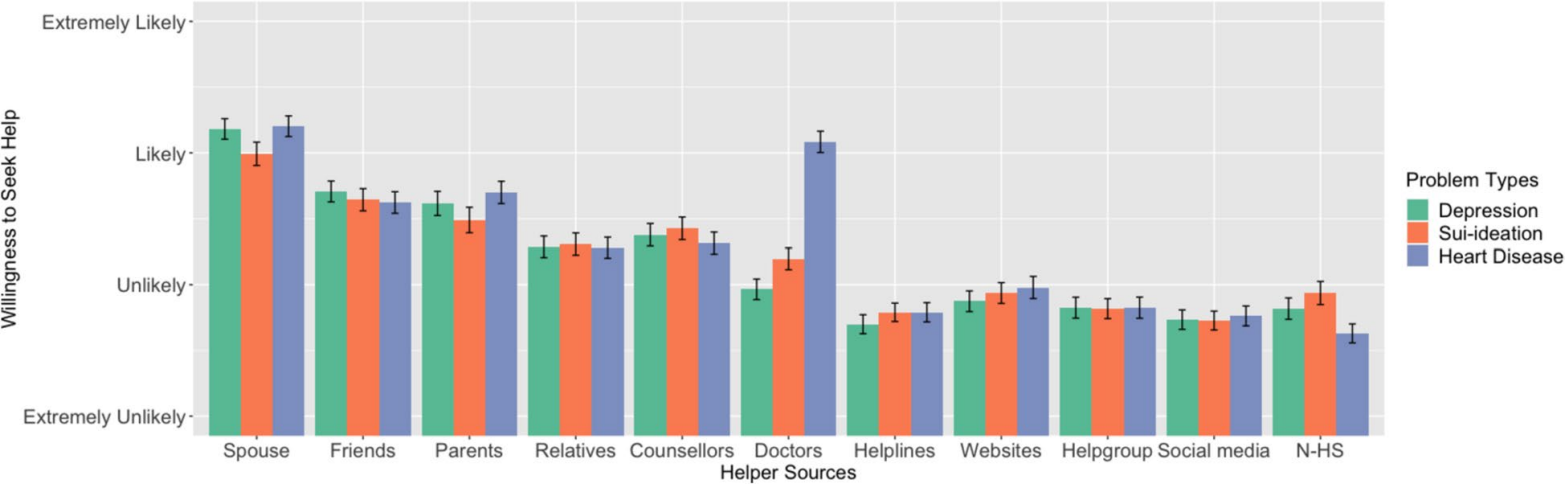
Help-seeking intentions across sources of help and problem types. *Notes*: For the two mental disorders, the “Doctors” source refers to “psychiatrists”, and for the heart disease, the “Doctors” source refers to “relevant doctors in hospital”. “Helpgroup” refers to online help groups such as internet forums. “N-HS” refers to “No Help-Seeking”, or “Won’t seek help” in the questionnaire

As shown in Fig. 1, there is a significant difference in the pattern of help-seeking intentions across three health conditions or problem types (depression, suicide ideation, and heart disease), *F*(2, 284) = 7.360, *p* = 0.001, η_p_^2^ = 0.049. Post-hoc analyses showed that while depressive symptoms and suicidal ideation did not differ significantly [*M*_depression-suicide ideation_ = -0.006, *SE* = 0.037, *p* = 0.997, Sidak adjusted 95% confidence interval for differences is (-0.095, 0.082)], they both elicited significantly lower willingness to seek help from participants than heart disease [*M*_depression-heart disease_ = -0.129, *SE* = 0.039, *p* = 0.004, Sidak adjusted 95% confidence interval for differences is (-0.225, -0.034); *M*_suicide ideation-heart disease_ = -0.123, *SE* = 0.038, *p* = 0.004, Sidak adjusted 95% confidence interval for differences is (-0.215, -0.031)]. Different help sources also significantly affect one’s help-seeking intention, *F*(9, 1278) = 72.646, *p* = 0.001, η_p_^2^ = 0.338. Among the ten sources, the willingness to seek help was decreasing from spouses to social media. Treating the different types of helpers as partially ordinal, the linear decreasing trend was highly significant, *F*(1,142) = 210.326, *p* < 0.001, η_p_^2^ = 0.597.

Importantly, the interaction between health conditions and help sources was significant, *F*(18, 2556) = 15.359, *p* < 0.001, η_p_^2^ = 0.098. Further simple effect analyses (Table 3) showed that for most helpers, participants generally showed no differences in willingness to seek help. However, people were slightly less likely to seek help from parents due to suicidal ideation compared with heart diseases; when facing doctors, people are significantly more likely to seek help for heart disease, then suicidal ideation, and depression the least.

**Table 3:** Simple effects following the two-way interaction between health conditions and helper sources

Helper Source	Health Conditions Comparisons (I-J)	Mean Difference (I-J)	*SE*	Sig	95% Confidence Interval for Difference	
					Lower Bound	Upper Bound
Spouse	Depression—Suicide Ideation	0.189	0.080	0.058	-0.004	0.382
	Depression—Heart Disease	-0.021	0.079	0.991	-0.212	0.170
	Suicide Ideation—Heart Disease	-0.210	0.089	0.057	-0.424	0.004
Friend	Depression—Suicide Ideation	0.063	0.069	0.741	-0.104	0.229
	Depression—Heart Disease	0.084	0.086	0.702	-0.124	0.292
	Suicide Ideation—Heart Disease	0.021	0.089	0.994	-0.194	0.236
Parents	Depression—Suicide Ideation	0.126	0.072	0.230	-0.048	0.300
	Depression—Heart Disease	-0.084	0.088	0.718	-0.298	0.130
	Suicide Ideation—Heart Disease	-0.210^*^	0.083	0.037	-0.410	-0.009
Relatives	Depression—Suicide Ideation	-0.021	0.070	0.987	-0.190	0.148
	Depression—Heart Disease	0.007	0.078	1.000	-0.183	0.197
	Suicide Ideation—Heart Disease	0.028	0.070	0.970	-0.141	0.197
Counsellors	Depression—Suicide Ideation	-0.049	0.074	0.881	-0.227	0.129
	Depression—Heart Disease	0.063	0.093	0.875	-0.162	0.288
	Suicide Ideation—Heart Disease	0.112	0.099	0.598	-0.128	0.352
Doctors	Depression—Suicide Ideation	-0.231^**^	0.077	0.009	-0.416	-0.046
	Depression—Heart Disease	-1.119^***^	0.104	< 0.001	-1.370	-0.868
	Suicide Ideation—Heart Disease	-0.888^***^	0.093	< 0.001	-1.113	-0.663
Helplines	Depression—Suicide Ideation	-0.091	0.068	0.455	-0.255	0.073
	Depression—Heart Disease	-0.091	0.072	0.507	-0.265	0.084
	Suicide Ideation—Heart Disease	<0.001	0.062	1.000	-0.150	0.150
Websites	Depression—Suicide Ideation	-0.063	0.068	0.735	-0.228	0.102
	Depression—Heart Disease	-0.105	0.071	0.372	-0.277	0.068
	Suicide Ideation—Heart Disease	-0.042	0.059	0.861	-0.186	0.102
Help-groups	Depression—Suicide Ideation	0.007	0.062	0.999	-0.142	0.156
	Depression—Heart Disease	<0.001	0.069	1.000	-0.168	0.168
	Suicide Ideation—Heart Disease	-0.007	0.051	0.999	-0.130	0.116
Social Media	Depression—Suicide Ideation	0.007	0.058	0.999	-0.134	0.148
	Depression—Heart Disease	-0.028	0.056	0.945	-0.164	0.108
	Suicide Ideation—Heart Disease	-0.035	0.048	0.849	-0.151	0.081

*p < 0.05, **p < 0.01 ***p < 0.001. Significance testing for multiple comparisons was adjusted using Sidak method

For the ANOVA on the intention of not seeking help, there is also a significant difference in the pattern of help-seeking intentions across three health conditions (depression, suicide ideation, and heart disease), *F*(2, 284) = 6.769, *p* = 0.001, η_p_^2^ = 0.046. Post-hoc analyses showed that people were least likely to not seek help for heart disease (*M* = 1.629, *SD* = 0.861), and were most likely to not seek help due to suicide ideation (*M* = 1.937, *SD* = 1.050). The difference between them was significant [*M*_suicide ideation – heart disease_ = 0.308, SE = 0.078, *p* < 0.001, Sidak adjusted 95% confidence interval for differences is (0.119, 0.497)]. The depressive symptoms (*M* = 1.818, *SD* = 0.081) ranked in the middle and did not differ significantly from the other two problem types [*M*_depression-heart disease_ = 0.189, *SE* = 0.088, *p* = 0.098, Sidak adjusted 95% confidence interval for differences is (-0.024, 0.402); *M*_depression – suicide ideation_ = -0.119, *SE* = 0.086, *p* = 0.429, Sidak adjusted 95% confidence interval for differences is (-0.327, 0.089)].

### Predictors of help-seeking intentions

As shown in Table 4, the condition results from Model 1 were similar to ANOVA findings (two significant main effects of Condition and of Helping sources; see above subsection), which revealed that from spouses to social media, the willingness to seek help was decreasing, and participants were more likely to seek help for heart disease. Among the predicting variables, participants’ self-efficacy level, educational levels, and suicide ideation could significantly predict one’s overall help-seeking intention. All other variables were not significant. Results from Model 2 showed that one’s suicide literacy could significantly predict a higher intention to not seek help.

**Table 4:** Predictors of help-seeking and no help-seeking intentions among rural residents

	Model 1: Help-seeking			Model 2: No help-seeking		
	Estimate	SE	*t*	Estimate	SE	*t*
*Fixed effect*						
Intercept	2.263	0.438	5.162***	1.718	0.632	2.719**
*Conditions (Reference* = *Heart Disease)*						
Problem-Depressive	-0.128	0.036	-3.559***	0.241	0.095	2.533*
Problem-Suicidal	-0.115	0.036	-3.198**	0.306	0.095	3.215**
*Help sources (Reference* = *Counselor)*						
Helper-Spouse	0.787	0.066	12.009***			
Helper-Friend	0.278	0.066	4.238***			
Helper-Parents	0.238	0.066	3.626***			
Helper-Relative	-0.062	0.066	-0.942			
Helper-Doctors	0.071	0.066	1.083			
Helper-Helplines	-0.565	0.066	-8.618***			
Helper-Websites	-0.392	0.066	-5.981***			
Helper-Help groups	-0.509	0.066	-7.770***			
Helper-Social Media	-0.620	0.066	-9.466***			
*“Need” Factors*						
Suicide literacy	-0.035	0.030	-1.181	0.090	0.043	2.072*
DQ-5 (Distress)	0.110	0.095	1.160	-0.105	0.138	-0.766
PHQ-9 (Depression)	-0.018	0.019	-0.971	-0.002	0.027	-0.074
GAD-7 (Anxiety)	-0.017	0.024	-0.701	0.043	0.035	1.217
Suicide exposure	-0.012	0.017	-0.724	0.038	0.024	1.570
Suicide ideation	0.715	0.210	3.402***	0.036	0.304	0.119
*“Enabling” Factors*						
Self-Efficacy	0.182	0.075	2.432*	0.009	0.108	0.082
*“Predisposing” Factors*						
Age	-0.003	0.004	-0.896	-0.005	0.005	-0.876
Educational levels	0.098	0.048	2.026*	-0.115	0.070	-1.657
Gender	-0.017	0.105	-0.159	-0.263	0.152	-1.727
*Variance Components*						
Intercept	0.225	0.036		0.356	0.076	
Residual	0.696	0.018		0.488	0.047	

*p < 0.05, **p < 0.01 ***p < 0.001. *DQ-5* Distress Questionnaire 5, *GAD-7* General Anxiety Disorder-7, *PHQ-9* Patient Health Questionnaire-9. PHQ-9, GAD-7 and suicide literacy were calculated as sum score, while DQ-5 and self-efficacy was mean score. Suicide exposure was calculated as a person’s highest level of exposure

Given that the results from Table 4 only showed the overall associations between various factors and help-seeking intentions, we also conducted separate mixed linear modeling to examine each condition (see Tables S1-S4 in Supplemental Files). The results^1^ are generally consistent with that of Table 4.

## Discussion

The present study investigated psychological help-seeking for suicidal ideation in a sample of 143 rural residents in 16 villages from four municipalities in central China, by conducting a field survey during home-visiting interviews. To our knowledge, the present study is the first to systematically inquire about the help-seeking patterns across various helping sources for mental health vs. physical conditions among rural residents in China. Besides, by employing GHSQ-V to measure help-seeking, the present study could provide a more ecologically valid picture of the rural residents’ help-seeking intentions as the health conditions were presented to respondents as symptoms rather than abstract medical terms as in previous studies.

Using ANOVA and linear mixed modeling, the results from the present study showed that in all three health symptomology conditions (symptoms of depression, suicide ideation, and heart disease), the pattern of help-seeking intentions regarding different helping sources is generally identical in rural residents; family, and friends were given top priority, then medical facilities, and internet channels were seen as the last resort. In other words, there is a general trend that the help-seeking intentions decrease as the helping sources shifted from one’s informal, close social circles to more distant, professional or nonprofessional ones. One notable exception to this trend is the preferable role of a doctor in the condition of heart disease, where rural residents prefer a doctor as the primary source, on a same level as one’s spouses, whereas doctors only ranked 6th—lower than counselors— in other two conditions. Besides, the results showed that the overall help-seeking intentions for suicide and depression were significantly lower than heart disease. Furthermore, while there are differences in specific conditions, the overall results from the linear mixed model suggested that rural residents with higher educational levels, higher self-efficacy, and severer suicide ideations were more willing to seek help; while the level of suicide literacy in the rural residents negatively predicted help-seeking intentions for all three health conditions.

Rural residents in the present study prefer family members and friends as their primary helping sources, formal channels such as doctors as their next set of choices, and internet channels as their least preferred sources. This preference pattern is in part consistent with past studies. In a recent study that targeted the help-seeking behaviors of the young generation in Hong Kong, researchers categorized the patterns of help-seeking behaviors of young people from Hong Kong via latent profile analysis [30]. The four types that emerged also reflected the gradation or the progression of social closeness from the close to the distant: non-seekers, inner circle seekers (i.e., family, friends, and partner), non-familial inner circle seekers (i.e., only seeking help from friends and partner ties), and diverse seekers; moreover, most of the participants in the study were categorized into either inner circle seekers or non-familial inner circle seekers, suggesting that for most people, family and friends are usually the first choice for help-seeking. Similarly, in a study involving Chinese mental disorder patients, the primary sources of their choice were family and friends, then medical facilities, and mental health facilities ranked only third [60]. This preference for alternative channels over professional institutions is often listed as one of the main obstacles precluding the Chinese population from seeking professional psychological help [20, 21, 23, 46].

Besides, the present study found that participants’ educational level, suicide ideation, and self-efficacy could all positively predict overall help-seeking intentions. It is generally believed that individuals with higher educational levels were less likely to be deterred by suicide or psychological help-seeking stigma, and would be more willing to seek psychological help should the need arise [12, 16]. In addition, participants with higher levels of suicide ideation may have encountered more stressors and experienced heightened psychological pain, both of which could lead to an elevated need for help. Individuals with higher self-efficacy are more confident about their action capabilities and thus may be better equipped with the agentic qualities and skill sets that are conducive to greater help-seeking intentions and behaviors [38]. The findings of the present study are generally consistent with previous research.

The present study also revealed a positive relationship between suicide literacy and the endorsement of “No Help-seeking”, contrary to most past literature on suicide literacy. In other words, for rural residents from the present sample, those with a better knowledge of suicide, its symptomology, prevention, as well as coping skills, exhibited a greater tendency not to seek any help, from any sources, and for all three health conditions (two of which bear no direct relation with suicide). Theoretically, one’s suicide literacy is negatively related to suicide stigma, and the related knowledge could facilitate a better understanding of the timing and ways for help-seeking; hence, suicide literacy should help increase help-seeking intentions [7, 63]. However, empirical research yielded mixed findings. Suicide literacy was associated with increased help-seeking intentions in one study [12], while in other studies such a relationship became non-significant [20, 21, 23]. Relatedly, a study from another line of research revealed a complex relationship between suicide literacy and the recommendation of professional help to others. In this study, people with higher suicide literacy were more likely to recommend informal sources in all cases other than suicide and only recommend professional helping sources to the target with manifestly suicide-related behaviors [18]. Therefore, contrary to the common theoretical view, the effect of suicide literacy on help-seeking for suicidal ideation and/or behavior could be more complex than previously assumed and could be subject to the influence of multiple factors.

The underlying causes for the negative association between suicide literacy and help-seeking intentions in the rural sample of the present study may be low accessibility to suicide intervention sources as well as rural residents’ generally low trust in medical facilities and counseling agencies [46], although direct studies on the potential causes are sparse. Despite the rapid development of the health system in recent years, China still suffers from a shortage of mental health institutions, and the availability and accessibility of mental health services are even lower in rural areas [39]. Added to the plight of rural residents is the poor coverage of health insurance in rural areas of China, which may have impeded the rural residents from seeking professional psychological help, particularly when they are knowledgeable enough to know the price tag and the time and effort it would take to access the services. Another possible cause may be the rural residents’ lack of trust in current mental health services and the overall suicide prevention system. It is possible that people with higher levels of suicide literacy may find the quality of help that their immediate circle (family or friends) or professional facilities nearby could provide unsatisfying and may thus refrain from seeking help from any of the sources. Currently, it remains to be seen if the link between suicide literacy and help-seeking reluctance could be replicated in other studies on rural residents from China or other developing countries, and further study is needed to examine the possible causes. Future studies may delve into the causes by combining qualitative and quantitative approaches, investigating rural residents’ perceptions of and emotional reactions to psychological helping sources, which may help explain the relationship between suicide literacy and help-seeking intentions.

The findings from the present study have several practical implications. First, the present findings that rural residents in China showed lower help-seeking intentions for psychological conditions (suicidal ideation and depression) than physical conditions (heart disease) again accentuated the urgent need for raising mental health awareness in rural communities. Campaigns for mental health awareness are recommended to address disparities in mental health services across urban and rural areas. Furthermore, given that the help-seeking pattern of rural residents usually favors close circles over professional and distant helpers, suicide prevention programs could capitalize on this tendency by fostering a tiered and tailored intervention network (e.g., a stepped care approach; [28]), in which an initial peer support and triage system could be strengthened and could cooperate effectively with professional mental health services. Implications for health policy may include gatekeeper training programs in rural communities, adding psychiatric screening to the annual medical checkup programs as well as primary care units in rural areas. In addition, related intervention programs should be recalibrated to meet the possible distrust from the rural residents, i.e., by implementing community outreach programs, or promoting the availability of services.

### Limitations

The present study has several limitations. First, the cross-sectional nature of the present study precluded a clear causal inference from the data. Future studies should employ longitudinal methodology and health intervention programs, such as internet interventions [20, 21, 23], to understand the effects of various factors that may have influenced help-seeking in rural areas. Second, as a largely exploratory study, the present study did not directly test the possible underlying psychological mechanisms of help-seeking. Future studies may further add more measures on the intermediate psychological variables and test the internal psychological mechanisms by mediation analysis or structural equation modeling (e.g., [34]). Third, the present study only examined help-seeking patterns in rural areas, and the nature of the dataset precluded a direct comparison between rural and urban residents (and other demographic groups) in the present analysis. A more systematic investigation of help-seeking behaviors across diverse social groups would be a fruitful avenue for future research.

## Conclusions

This study provides the first evidence of the patterns and predictors of help-seeking intentions for suicidal ideation, mental health (depression), and physical health (heart disease) conditions among Chinese rural residents. Across all health conditions, family and friends were the top preference for help-seeking, followed by medical professionals and internet-based resources. Besides, rural residents showed generally lower help-seeking intentions for depression and suicidal ideation than heart disease, and the rural residents with higher educational levels, greater self-efficacy, and more severe suicidal ideations were more likely to seek help. However, higher suicide literacy was negatively associated with help-seeking intentions. Several other variables, including suicide exposure, respondents’ age, gender, their current level of psychological distress, depression, and anxiety, all failed to predict help-seeking intentions. Overall, the findings highlight the need to develop a stepped-care model for suicide prevention tailored to the Chinese population.

## Supplementary Information

Supplementary Material 1.

## Data Availability

The data supporting this study's findings are not openly available due to reasons of sensitivity and the deidentified version of the dataset is available from the corresponding author upon reasonable request.

## Footnotes

1 The results from separate mixed linear models for Depression, Suicidal ideation and Heart disease are generally consistent with the overall model, with only minor disparities. One is that the effect of self-efficacy was only marginally significant in predicting help-seeking in suicidal ideation (Table S2 in Supplemental Files) and heart disease (Table S3 in Supplemental Files); another is that the effect of educational level was not significant in predicting help-seeking in Depression (Table S1 in Supplemental Files). To test whether the effects of these predictors may differ across health conditions require a moderation analysis, and the limited sample size of the present study could not provide enough power to test the potential moderation effect.
