# Supplementary material for: Patterns and predictors of help-seeking intentions for suicidal ideation compared to other health conditions among rural Chinese adults
Source: BMC Psychiatry. 2024 Oct 24;24:727. doi: 10.1186/s12888-024-06186-0 (PMC11515480; doi:10.1186/s12888-024-06186-0)
Supplement: Supplementary file 1 — Supplementary Material 1. [file 12888_2024_6186_MOESM1_ESM.docx]

Table S1.

*Depression*

|  | Estimate | SE | *t* |
| --- | --- | --- | --- |
| *Fixed effect* |  |  |  |
| Intercept | 2.200 | 0.473 | 4.655*** |
| *Help sources (Reference = Counselor)* | | |  |
| Helper-Spouse | 0.833 | 0.112 | 7.464*** |
| Helper-Friend | 0.343 | 0.112 | 3.069** |
| Helper-Parents | 0.231 | 0.112 | 2.073* |
| Helper-Relative | -0.046 | 0.112 | -0.415 |
| Helper-Doctors | -0.398 | 0.112 | -3.566*** |
| Helper-Helplines | -0.648 | 0.112 | -5.806*** |
| Helper-Websites | -0.435 | 0.112 | -3.898*** |
| Helper-Help groups | -0.537 | 0.112 | -4.810*** |
| Helper-Social Media | -0.648 | 0.112 | -5.806*** |
| *“Need” Factors* | |  |  |
| Suicide literacy | -0.026 | 0.032 | -0.821 |
| DQ-5 (Distress) | 0.117 | 0.102 | 1.152 |
| PHQ-9 (Depression) | -0.015 | 0.020 | -0.759 |
| GAD-7 (Anxiety) | -0.021 | 0.026 | -0.802 |
| Suicide exposure | -0.008 | 0.018 | -0.434 |
| Suicide ideation | 0.751 | 0.225 | 3.338** |
| *“Enabling” Factors* | |  |  |
| Self-Efficacy | 0.221 | 0.080 | 2.762** |
| *“Predisposing” Factors* | |  |  |
| Age | -0.003 | 0.004 | -0.840 |
| Educational levels | 0.016 | 0.052 | 0.314 |
| Gender | -0.011 | 0.113 | -0.097 |
| *Variance Components* | |  |  |
| Intercept | 0.217 | 0.041 |  |
| Residual | 0.673 | 0.031 |  |

Table S2

*Suicide*

|  | Estimate | SE | *t* |
| --- | --- | --- | --- |
| *Fixed effect* |  |  |  |
| Intercept | 2.384 | 0.537 | 4.436*** |
| *Help sources (Reference = Counselor)* | | |  |
| Helper-Spouse | 0.630 | 0.106 | 5.932*** |
| Helper-Friend | 0.231 | 0.106 | 2.181* |
| Helper-Parents | 0.120 | 0.106 | 1.134 |
| Helper-Relative | -0.093 | 0.106 | -0.872 |
| Helper-Doctors | -0.185 | 0.106 | -1.745† |
| Helper-Helplines | -0.528 | 0.106 | -4.972*** |
| Helper-Websites | -0.426 | 0.106 | -4.013*** |
| Helper-Help groups | -0.537 | 0.106 | -5.059*** |
| Helper-Social Media | -0.667 | 0.106 | -6.281*** |
| *“Need” Factors* | |  |  |
| Suicide literacy | -0.058 | 0.037 | -1.580 |
| DQ-5 (Distress) | 0.071 | 0.116 | 0.610 |
| PHQ-9 (Depression) | -0.024 | 0.023 | -1.067 |
| GAD-7 (Anxiety) | -0.014 | 0.030 | -0.471 |
| Suicide exposure | -0.013 | 0.021 | -0.654 |
| Suicide ideation | 0.784 | 0.257 | 3.053** |
| *“Enabling” Factors* | |  |  |
| Self-Efficacy | 0.168 | 0.092 | 1.834† |
| *“Predisposing” Factors* | |  |  |
| Age | -0.005 | 0.005 | -1.004 |
| Educational levels | 0.121 | 0.059 | 2.056* |
| Gender | 0.048 | 0.129 | 0.375 |
| *Variance Components* | |  |  |
| Intercept | 0.310 | 0.053 |  |
| Residual | 0.608 | 0.028 |  |

Table S3

*Heart Disease*

|  | Estimate | SE | *t* |
| --- | --- | --- | --- |
| *Fixed effect* |  |  |  |
| Intercept | 1.964 | 0.491 | 4.000*** |
| *Help sources (Reference = Counselor)* | | |  |
| Helper-Spouse | 0.898 | 0.110 | 8.180*** |
| Helper-Friend | 0.259 | 0.110 | 2.361* |
| Helper-Parents | 0.361 | 0.110 | 3.289** |
| Helper-Relative | -0.046 | 0.110 | -0.422 |
| Helper-Doctors | 0.796 | 0.110 | 7.252*** |
| Helper-Helplines | -0.519 | 0.110 | -4.722*** |
| Helper-Websites | -0.315 | 0.110 | -2.867** |
| Helper-Help groups | -0.454 | 0.110 | -4.132*** |
| Helper-Social Media | -0.546 | 0.110 | -4.975*** |
| *“Need” Factors* | |  |  |
| Suicide literacy | -0.022 | 0.033 | -0.657 |
| DQ-5 (Distress) | 0.143 | 0.106 | 1.347 |
| PHQ-9 (Depression) | -0.015 | 0.021 | -0.714 |
| GAD-7 (Anxiety) | -0.016 | 0.027 | -0.600 |
| Suicide exposure | -0.015 | 0.019 | -0.815 |
| Suicide ideation | 0.610 | 0.234 | 2.605* |
| *“Enabling” Factors* | |  |  |
| Self-Efficacy | 0.157 | 0.083 | 1.882† |
| *“Predisposing” Factors* | |  |  |
| Age | -0.002 | 0.004 | -0.504 |
| Educational levels | 0.156 | 0.054 | 2.900** |
| Gender | -0.088 | 0.117 | -0.747 |
| *Variance Components* | |  |  |
| Intercept | 0.243 | 0.044 |  |
| Residual | 0.651 | 0.030 |  |

Table S4

*Predictor: No help-seeking*

|  | Depression | | | Suicide | | | Heart Disease | | |
| --- | --- | --- | --- | --- | --- | --- | --- | --- | --- |
|  | Estimate | SE | *t* | Estimate | SE | *t* | Estimate | SE | *t* |
| *Coefficients* |  |  |  |  |  |  |  |  |  |
| Intercept | 1.385 | 0.854 | 1.622 | 2.161 | 0.840 | 2.574* | 2.154 | 0.696 | 3.093** |
| *“Need” Factors* | |  |  |  |  |  |  |  |  |
| Suicide literacy | 0.075 | 0.059 | 1.274 | 0.167 | 0.058 | 2.889** | 0.028 | 0.048 | 0.574 |
| DQ-5 (Distress) | -0.225 | 0.187 | -1.208 | -0.082 | 0.184 | -0.447 | -0.009 | 0.152 | -0.057 |
| PHQ-9 (Depression) | 0.006 | 0.037 | 0.157 | -0.005 | 0.036 | -0.139 | -0.007 | 0.030 | -0.225 |
| GAD-7 (Anxiety) | 0.069 | 0.048 | 1.458 | 0.028 | 0.047 | 0.594 | 0.031 | 0.039 | 0.796 |
| Suicide exposure | 0.017 | 0.033 | 0.505 | 0.063 | 0.032 | 1.952† | 0.035 | 0.027 | 1.285 |
| Suicide ideation | -0.148 | 0.412 | -0.359 | 0.011 | 0.405 | 0.027 | 0.245 | 0.336 | 0.730 |
| *“Enabling” Factors* | |  |  |  |  |  |  |  |  |
| Self-Efficacy | 0.159 | 0.147 | 1.084 | -0.090 | 0.144 | -0.627 | -0.042 | 0.120 | -0.351 |
| *“Predisposing” Factors* | |  |  |  |  |  |  |  |  |
| Age | -0.001 | 0.007 | -0.191 | -0.006 | 0.007 | -0.838 | -0.007 | 0.006 | -1.130 |
| Educational levels | -0.189 | 0.095 | -2.002* | -0.107 | 0.093 | -1.148 | -0.050 | 0.077 | -0.655 |
| Gender | -0.149 | 0.207 | -0.720 | -0.346 | 0.203 | -1.702† | -0.295 | 0.169 | -1.749† |
|  |  |  |  |  |  |  |  |  |  |
| *R^2^* | 0.108 |  |  | 0.192 |  |  | 0.099 |  |  |
| *F*(10, 97) = 1.170, *p* =0.321 | |  |  | *F*(10, 97) = 2.308, *p* =0.018 | | | *F*(10, 97) = 1.063, *p* =0.398 | | |
